# C‐type lectin domain group 14 proteins in vascular biology, cancer and inflammation

**DOI:** 10.1111/febs.14985

**Published:** 2019-07-29

**Authors:** Kabir A. Khan, Jack L. McMurray, Fiyaz Mohammed, Roy Bicknell

**Affiliations:** ^1^ Biological Sciences Platform Sunnybrook Research Institute Toronto Canada; ^2^ Department of Medical Biophysics University of Toronto Canada; ^3^ Cancer Immunology and Immunotherapy Centre Institute of Immunology and Immunotherapy University of Birmingham UK; ^4^ Institutes of Cardiovascular Sciences and Biomedical Research College of Medical and Dental Sciences University of Birmingham UK

**Keywords:** cancer, CD248, CD93, CLEC14A, C‐type lectin, extracellular matrix, group XIV, immuno‐oncology, thrombomodulin, vascular targeting

## Abstract

The C‐type lectin domain (CTLD) group 14 family of transmembrane glycoproteins consist of thrombomodulin, CD93, CLEC14A and CD248 (endosialin or tumour endothelial marker‐1). These cell surface proteins exhibit similar ectodomain architecture and yet mediate a diverse range of cellular functions, including but not restricted to angiogenesis, inflammation and cell adhesion. Thrombomodulin, CD93 and CLEC14A can be expressed by endothelial cells, whereas CD248 is expressed by vasculature associated pericytes, activated fibroblasts and tumour cells among other cell types. In this article, we review the current literature of these family members including their expression profiles, interacting partners, as well as established and speculated functions. We focus primarily on their roles in the vasculature and inflammation as well as their contributions to tumour immunology. The CTLD group 14 family shares several characteristic features including their ability to be proteolytically cleaved and engagement of some shared extracellular matrix ligands. Each family member has strong links to tumour development and in particular CD93, CLEC14A and CD248 have been proposed as attractive candidate targets for cancer therapy.

## Graphical abstract

The C‐type lectin domain‐containing group 14 family consists of thrombomodulin, CD248 (endosialin), CD93 and CLEC14A. These proteins exhibit a wide range of different functions in vascular biology, cancer and inflammation. In this review, we present and discuss what is known so far for each molecule and where current gaps in knowledge exist.

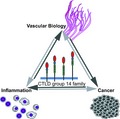

**Abbreviations**
ADAM10: a disintegrin and metalloproteinase‐10
ADC: antibody‐drug conjugate
CHO: Chinese hamster ovary
CTLD: C‐type lectin domain
ECD: extracellular domain
EGF: epidermal growth factor
EGFR1: epidermal growth factor receptor‐1
EMT: epithelial mesenchymal transition
EPCs: endothelial progenitor cells
ERK: extracellular‐signal regulated kinase
ERM: ezrin‐radixin‐moesin
GPR15: G protein‐coupled receptor‐15
GVHD: graft versus host disease
HCC: hepatocellular carcinoma
HMGB1: high‐mobility group protein B1
HUVEC: human umbilical vein endothelial cells
LLCs: Lewis lung carcinomas
LPS: lipopolysaccharide
MCAM: melanoma cell adhesion molecule
MMP9: matrix metalloproteinase‐9
MMRN2: multimerin‐2
NSCLC: nonsmall cell lung cancer
PDGF: platelet‐derived growth factor
PI3K: phosphoinositide 3‐kinase
RHBDL2: rhomboid like 2
TEM-1: tumour endothelial marker 1
TNFα: tumour necrosis factor‐α
VEGF: vascular endothelial growth factor
VEGFR2: vascular endothelial growth factor receptor 2
VSMCs: vascular smooth muscle cells

## Introduction: C‐type lectin domain group 14 family

There are 17 families in the C‐type lectin domain (CTLD) containing superfamily described in humans. This superfamily comprises a range of remarkably diverse proteins that can be secreted or expressed on the cell surface. They mediate a wide range of functions including but not limited to inflammation, cell adhesion and carbohydrate recognition 1.

**Figure 1:**
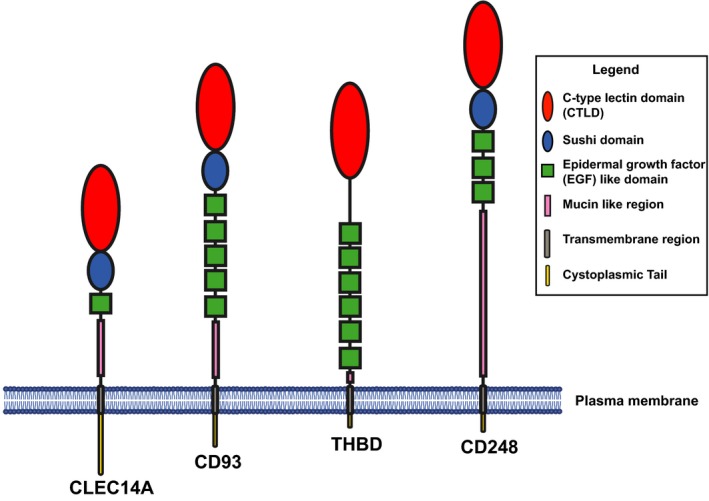
CTLD group 14 family proteins. Schematic diagrams of the CTLD group 14 family proteins. Each protein is drawn to relative scale based on primary amino acid sequence length. The CTLD is shown in red, the sushi in blue and the EGF repeats in green.

Thrombomodulin, CD248, CD93 and CLEC14A represent members of the CTLD group 14 family which share common domain architecture (Fig. 1). Each member is comprised of an N‐terminal signal peptide and a CTLD containing eight conserved cysteine residues. This is followed by a sushi‐like or complement control protein domain (also commonly referred to as a short consensus repeat), except for thrombomodulin which due to a lack of four conserved cysteine residues in this region does not satisfy the requirement for a sushi domain. Next are a number of EGF‐like domain repeats, thrombomodulin contains six, CD93 five, CD248 three and CLEC14A one. These are followed by a mucin‐like region of variable length which is proline, serine and threonine rich and encompasses many predicted O‐linked glycosylation sites. Finally, there is a single‐pass transmembrane region that connects to a cytoplasmic tail.

The CTLD was originally described as a calcium (Ca^2+^)‐dependent carbohydrate binding domain, although not all CTLDs require Ca^2+^ or demonstrate carbohydrate binding activity. The overall CTLD‐fold is characterised by a so called ‘loop in a loop’ structure stabilised by a conserved set of residues which contribute to a distinctive hydrophobic core 1. CTLD containing proteins have been widely described in many species and can even be found in the *Bordetella bronchiseptica* bacteriophage 2. Sushi domains exhibit extensive sequence variation but are generally characterised by four conserved cysteines (forming two disulfide linkages in a 1–4 and 2–3 pattern) and an invariant tryptophan, which contribute to preserving its tertiary structure 3. The sushi domain is an extracellular motif that can contribute to protein–protein interactions, best exemplified in interleukin‐15 receptor‐α (IL‐15Rα) recognition of IL‐15 4. EGF‐like domains are evolutionary conserved modules, which derive their name from the epidermal growth factor where they were originally described. EGF‐like domains are found in a wide range of proteins, chiefly of animal origin and are frequently observed in tandem repeats. Each EGF module typically consists of 30–40 amino acids and includes six conserved cysteines which form three intramolecular disulfide bonds 5. The highly glycosylated mucin region is commonly associated with adhesion proteins as described for CD164 6 and offers protection against protein degradation by preventing access to proteases. In addition, the presence of many O‐linked sugar moieties most likely allows proteins to adopt a more rigid and extended conformation 7. All of the CTLD group 14 family members have been detected at a much higher molecular weight than one would expect based on their primary amino acid sequences. These apparent disparities can be attributed to high degrees of post‐translational modifications, most likely glycosylation. Consistent with this hypothesis, when CD248 is treated with O‐glycanase and sialase, its molecular weight is reduced from 165 to 95 kDa when purified from human neuroblastoma cells 8. Similar findings have been reported when CD93 is treated with enzymes that remove O‐glycosylation 9. It is interesting to note that electron microscopy analysis of thrombomodulin revealed an elongated molecule with a large globular nodule at one end and a smaller nodule at the other 10. If we assume that the larger nodule is likely the CTLD, the smaller one is most likely comprised of the EGF repeats. Since the overall domain architecture of CTLD group 14 family members is relatively conserved, it is tempting to speculate that they all display a similar elongated structure with the membrane‐distal CTLD interacting with its cognate ligands. Additionally, the domain layout of CTLD, sushi and EGF modules are reminiscent of the CTLD group 4 selectin family of cell adhesion molecules, albeit in a different order 11. Similar to the group 4 family, there are numerous examples of the CTLD group 14 family mediating roles in adhesion (see below).

**Figure 2:**
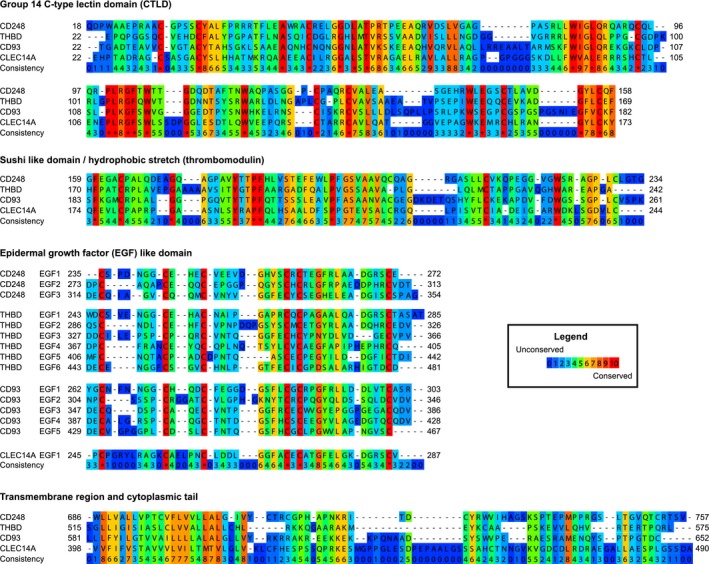
Alignments of CTLD group 14 family members based on amino acid sequence. Amino acid alignments of the whole primary sequence of each human family member using PRALINE 229. The following protein sequences were used thrombomodulin (http://www.uniprot.org/uniprot/P07204), CD93 (http://www.uniprot.org/uniprot/Q9NPY3), CLEC14A (http://www.uniprot.org/uniprot/Q86T13) and CD248 (http://www.uniprot.org/uniprot/Q9HCU0).

Based on whole protein sequence alignment, the family member with closest homology to CLEC14A is CD248 and CD93 is most closely related to thrombomodulin (Fig. 2). It has been suggested that CD93 could have arisen from thrombomodulin by gene duplication events as each is present on chromosome 20 in humans 12.

## Thrombomodulin

Thrombomodulin (THBD or CD141) is the most extensively studied member of the CTLD group 14 family and is expressed by endothelium of all blood vessels and lymphatics 12, 13. It is also localised on a range of other cell types including but not restricted to monocytes, neutrophils and dendritic cells 14. Thrombomodulin is expressed early in development and mice lacking the gene show embryonic lethality 15. Interestingly, thrombomodulin‐deficient mouse embryos die at embryonic day 8.5 due to defects in nonendothelial tissue within the placenta, but reintroduction of thrombomodulin into the placenta allows normal development of embryos until day 12.5 16. This suggests two distinct roles for thrombomodulin during development, one in the placenta and the other in the embryo. Nevertheless, thrombomodulin is the only family member that following genetic deletion causes embryonic lethality, suggesting that it exhibits an indispensable role. This lethal phenotype is not dependent on the CTLD or the cytoplasmic tail, as mice that lack these modules remain viable 17, 18. Based on these considerations, such embryonic lethality is most likely due to disruptions in the thrombomodulin‐mediated coagulation cascade elicited by the EGF domain tandem repeats (see below).

## Thrombomodulin and coagulation

One of the major roles for thrombomodulin is regulating the coagulation cascade by binding to the serine protease thrombin 19. The mode of recognition for this physiologically relevant co‐factor involves the EGF modules of thrombomodulin as determined by the crystal structure of the thrombomodulin–thrombin complex 20. This binding event inhibits procoagulant thrombin‐mediated hydrolysis of fibrinogen to fibrin, thereby inducing an anticoagulative effect 21. Thrombomodulin–thrombin binding also increases by approximately 1000‐fold the thrombin‐mediated cleavage and activation of the anticoagulant serine protease protein C 19. Activated protein C is involved in the inactivation of procoagulant factors FVa and FVIIIa 19, 22. In addition, thrombomodulin–thrombin complexes enhanced by approximately 1250‐fold the activation of the antifibrinolytic TAFI (thrombin activatable fibrinolysis inhibitor) 23. Therefore, by redirecting its cleavage activity towards the activation of anticoagulant and antifibrinolytic proteins, thrombomodulin can dampen the coagulation cascade by different mechanisms. Recently, the regulators of angiogenesis angiopoietin‐1 (Ang‐1) and angiopoietin‐2 (Ang‐2) have both been described as ligands for thrombomodulin *in vitro* 24. Ang‐2 binds with higher affinity than Ang‐1 but both, by competing with thrombin, can disrupt thrombomodulin–thrombin interactions leading to suppression of thrombin‐mediated anticoagulant functions. Thrombomodulin binding to heat shock protein 70 (HSP70‐1) on the endothelial cell surface can also inhibit thrombomodulin function *in vitro* by reducing protein C activation by as yet unknown mechanisms 25. The wide‐ranging roles of thrombomodulin in coagulation are well‐documented elsewhere 24, 26 and hence will not be discussed in any depth. We also direct the reader towards a recent extensive review exploring the ‘nontraditional roles’ of thrombomodulin 27.

## Thrombomodulin and angiogenesis

Proangiogenic effects have been reported for a recombinant form of soluble thrombomodulin encompassing six contiguous EGF modules and the mucin‐like region (thrombomodulin^EGF‐Mucin^), resulting in increased endothelial proliferation, tube formation, migration and upregulation of matrix metalloprotease expression *in vitro* 28. This recombinant protein also elicited proangiogenic effects on endothelial progenitor cells (EPCs) through a phosphoinositide 3‐kinase (PI3K)‐dependent pathway 29. Furthermore, thrombomodulin^EGF‐Mucin^ demonstrated endothelial protective roles chiefly by guarding against apoptosis again via the PI3K pathway 30. These roles are thought to be dependent on the EGF domains which can bind to and activate fibroblast growth factor receptor 1 (FGFR1) 31. The fifth EGF domain of thrombomodulin alone (thrombomodulin^EGF5^) has also demonstrated proangiogenic function as well as cytoprotective effects on endothelium 32. This cytoprotective phenomenon was suggested to be independent of thrombomodulin–thrombin interactions and instead due to upregulation of antiapoptotic protein myeloid‐cell leukaemia‐1 (MCL1) 33. A subsequent study revealed that this cytoprotective outcome was triggered by thrombomodulin^EGF5^ binding to G protein‐coupled receptor‐15 (GPR15) on endothelial cells, leading to the activation of endothelial nitric oxide synthase and extracellular signal‐regulated kinase (ERK) signalling, an effect that was abolished in GPR15‐deficient mice 34. Recently, the minimal fragment of thrombomodulin^EGF5^ necessary for binding to GPR15 and promoting proangiogenic function was identified as a 19‐amino acid peptide, that includes an intramolecular disulfide bond which adopts a loop structure similar to that observed for the prototypical EGF 31, 35. This peptide exhibited proangiogenic function and extended survival in mouse models of sinusoidal obstruction syndrome, a condition that is associated with injury of liver sinusoidal endothelium 35. However, whether thrombomodulin can bind to GPR15 while attached to the cell membrane, or if proteolytic processing is essential, is yet to be determined.

These proangiogenic signals mediated by the thrombomodulin EGF5 domain can be abolished when the soluble extracellular domain (ECD) contains the CTLD 36. The CTLD of thrombomodulin binds to Lewis Y antigen, which is a cell surface tetrasaccharide that is predominantly expressed during development and tumourigenesis 37. Relatedly, soluble CTLD alone can mediate aberrant effects in angiogenesis assays, presumably by virtue of its interactions with Lewis Y antigen localised on epidermal growth factor receptor‐1 (EGFR1), thereby inhibiting its activation 36. These findings suggest that the thrombomodulin CTLD exhibits roles distinct from the EGF domains and may be functionally dominant in its soluble form, due to its ability to negate EGF domain‐dependent effects. The CTLD of membrane‐bound thrombomodulin has been shown to bind to the extracellular matrix protein fibronectin, an interaction which activates focal adhesion kinase phosphorylation and upregulates matrix metalloproteinase‐9 (MMP9) 37, 38. The thrombomodulin–fibronectin interaction occurs on tumour blood vessels in murine melanoma suggesting that this interplay may serve as a putative target for antiangiogenic therapy, although an in‐depth understanding of this interaction in healthy tissues would first need to be considered. Thrombomodulin cell surface expression can be regulated by binding of the CTLD to Kringle 1–5, a proteolytically cleaved fragment of plasminogen 39. This binding event results in thrombomodulin internalisation and degradation, negating the proangiogenic roles of membrane‐bound thrombomodulin.

## Thrombomodulin and cancer

Thrombomodulin expression has been described in multiple cancer types on the endothelium and tumour cells 40, 41. In genetically engineered mice expressing a mutant form of thrombomodulin with severely compromised thrombin binding, primary tumour growth was unaffected whereas lung metastasis was significantly enhanced 42. The authors suggested this observation was due to prolonged survival of tumour cells in the lung and demonstrated that this effect was attributed to the thrombin binding function and not the N‐terminal CTLD. A whole host of studies in different tumour settings (lung, colorectal, cervical, prostate and bladder) postulate a role for thrombomodulin overexpression in reversing epithelial mesenchymal transition (EMT) 43, 44, 45, 46, 47, 48. Upregulation of thrombomodulin may even enhance tumour sensitivity to chemotherapeutic agents, such as doxorubicin 49. Indeed, a more comprehensive review of the role of thrombomodulin in tumour biology has been documented 40. The overall findings seem to indicate that thrombomodulin expression correlates with a good prognosis and expression is abolished in more aggressive and highly metastatic tumour types.

More recently, soluble human thrombomodulin has been utilised as a potential cancer therapeutic agent and reductions in tumour growth were observed when administered to mice bearing pancreatic tumour xenografts 50. Furthermore, the soluble ECD of thrombomodulin has also been reported to reduce tumour growth in inflamed models of gastrointestinal tumours 51. Whether soluble thrombomodulin could have antitumour effects in patients has not been formally investigated; however, it has demonstrated clinical benefits in managing disseminated intravascular coagulation in cancer patients and has the potential for direct effects on tumour burden as well as in aberrant thrombosis 52.

## Thrombomodulin and inflammation

Thrombomodulin has been described to have roles in inflammation some of which are linked to its anticoagulant function. This is best exemplified by protein C triggering an anti‐inflammatory signalling cascade by inhibiting tumour necrosis factor‐α (TNFα) production in response to lipopolysaccharide (LPS) 53. Independent of its roles in coagulation inhibition, thrombomodulin CTLD can bind to the proinflammatory molecule high‐mobility group protein B1 (HMGB1), leading to suppression of inflammation *in vivo* and protection against LPS‐induced lethality 54. A more recent study highlighted that the thrombomodulin–HMGB1 interaction allows the EGF domain bound thrombin to proteolytically cleave HMGB1 55. The inactivation of HMGB1 has potential implications on immunogenic cell death events following anticancer intervention invoked by chemotherapeutic agents or radiotherapy, whereby HMGB1 released by dying cells serves as damage‐associated molecular patterns activating antigen‐presenting cells and facilitating presentation of tumour antigens 56. The thrombomodulin CTLD has also been shown to reduce the adhesion of polymorphonuclear leucocytes to endothelium 57. The authors proposed that this process was dependent on thrombomodulin binding to Lewis Y antigen and therefore blocking its availability to bind leucocytes and aid subsequent transmigration. Conversely, human leucocytes have been suggested to directly recognise the mucin‐like region of thrombomodulin through leucocyte adhesion integrins lymphocyte function‐associated antigen‐1 (LFA‐1) and Mac‐1 *in vitro* 58. Thrombomodulin EGF domains were found to markedly suppress LPS‐induced inflammatory signalling pathways in macrophages by associating with the pattern recognition receptor CD14, and that this inhibitory effect was also dependent on the serine threonine‐rich domain 59.

Thrombomodulin^EGF5^ also has the capacity to engage T‐cells bearing GPR15 60. This results in immunosuppression of T‐cell responses and facilitates the differentiation of regulatory T‐cells (T_regs_). In addition, recombinant thrombomodulin^EGF5^ was shown to inhibit dendritic cell activation. Taken together, this provides a possible rationale for recombinant thrombomodulin‐mediated alleviation of graft versus host disease (GVHD) in mouse models of haematopoietic stem cell transplantation 32. More importantly, it reconciles with clinical observations that increased expression of thrombomodulin can reduce GVHD in patients 32, 61. This immunosuppressive role of thrombomodulin could also have relevance in tumour immunology as thrombomodulin expressed in the tumour microenvironment has the potential to expand anti‐inflammatory and protumour T_regs_, a cell type that contributes to tumour immune evasion mechanisms 62. However, these findings are contrary to observations reporting thrombomodulin as a good prognostic factor, and its role in immunosuppression may be outweighed by its function in EMT and aggressiveness. Thrombomodulin also exhibits anti‐inflammatory properties by regulating the alternative pathway of complement activation. Complement activation is a critical process in inflammation of the innate immune system and involves the cleavage of various proteins resulting in proteolytic release of chemotactic factors and activation of further cleavage events ultimately leading to the assembly of pathogen destruction complexes 63. Thrombomodulin–thrombin‐mediated activation of TAFI cleaves complement proteins C3a and C5a leading to their inactivation 64 which has been demonstrated both *in vitro* and *in vivo* 65, 66. Thrombomodulin binds to complement protein C3b and enhances its factor H‐ and factor I‐mediated degradation into its inactive form (iC3b), another mechanism of negative regulation of the alternative complement pathway 67.

## Thrombomodulin shedding

There are many transmembrane proteins that are specifically shed from the cell membrane to activate or deactivate distinct protein functions in angiogenesis and other physiological processes. Examples include the membrane‐bound EGF precursor proteins, which are cleaved by metalloproteinases such as ADAM10 (a disintegrin and metalloproteinase‐10) and ADAM17 resulting in growth factor activation 68. Conversely, the EGF receptor itself can be subjected to proteolytic cleavage thereby suppressing downstream signalling functions 69.

Thrombomodulin can be cleaved by the serine protease rhomboid‐like 2 (RHBDL2) at a site proximal to the transmembrane domain, resulting in release of the entire ECD 70. This RHBDL2 cleaved form of thrombomodulin can increase migration and wound healing in keratinocytes *in vitro* 71. Also, the full‐length thrombomodulin‐ECD has also been shown to be cleaved from the endothelial cell surface after incubation with the neutrophil proteases elastase, cathepsin G and proteinase 3 72. Soluble thrombomodulin has been detected in human blood, urine and synovial fluid 73, 74, 75. Indeed, monitoring serum levels of soluble thrombomodulin may be important as it can positively correlate with disease status such as systemic lupus erythematosus 76.

The thrombomodulin CTLD can also be cleaved leaving the remainder of the molecule intact upon the cell surface, an event that is most likely facilitated by matrix metalloproteinases (MMPs) 77. Although, these cleavage events are yet to be explicitly shown *in vivo*, it is noteworthy that two forms of thrombomodulin have been isolated from human urine 74. Characterisation of these fragments by N‐terminal sequencing revealed that one form encompasses the EGF repeats and the mucin‐rich region and retained the ability to bind thrombin. In contrast, the second fragment corresponded to the equivalent molecular weight for the N‐terminal CTLD and failed to bind thrombin. Furthermore, four different versions of thrombomodulin were detected in human plasma suggesting even more potential cleavage sites 78. Indeed, the physiological relevance of these different fragments requires further investigation. Although not resulting in shedding, neutrophil proteases have been shown to inactivate human thrombomodulin by oxidation of a key methionine between EGF4 and EGF5 79.

In summary, these findings of differential shedding along with the distinct biological function of each thrombomodulin subdomain offers a scenario where one molecule can be proteolytically processed in different ways to elicit opposing effects. The shedding of thrombomodulin is likely a tightly regulated process in which specific domains are released depending on the requirement for pro or antiangiogenic signals, as well as pro or anti‐inflammatory outcomes.

## Additional roles for thrombomodulin

Along with its vital roles in regulating blood coagulation and inflammation, thrombomodulin also reportedly contributes to cell–cell adhesion *in vitro*, an event which is dependent upon the CTLD 80. This proadhesion role was Ca^2+^ dependent and could be abolished with CTLD‐specific antibodies or addition of mannose, chondroitin sulfate A or C. This suggests that the thrombomodulin CTLD serves as a conventional Ca^2+^‐dependent carbohydrate‐binding lectin.

**Figure 3:**
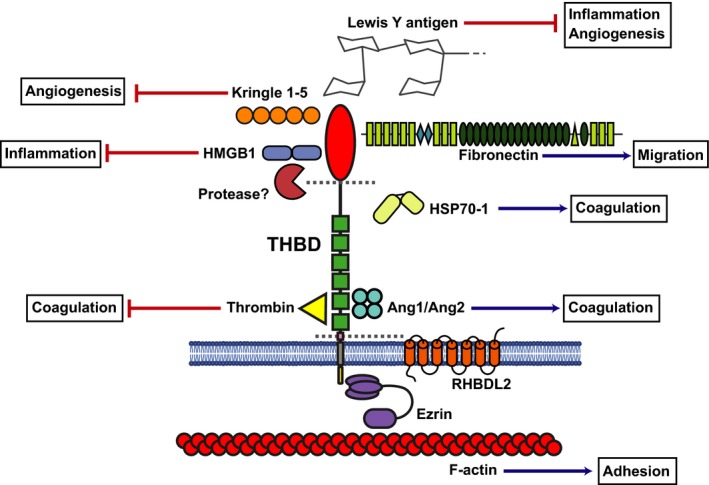
Schematic of thrombomodulin protein structure with ligand binding partners. Thrombomodulin CTLD has been shown to interact with fibronectin, HMGB1, Kringle 1–5, Lewis Y antigen and HSP70‐1. The CTLD may be proteolytically cleaved by an as yet unidentified MMP. Thrombin binds to the 5th and 6th EGF domains, this binding is in competition with Ang1 and/or Ang2. RHBDL2 can cleave the whole ECD of thrombomodulin as can neutrophil elastase, cathepsin G and proteinase 3. The cytoplasmic tail binds to ezrin which in turn links thrombomodulin to the actin cytoskeleton.

The cytoplasmic tail of thrombomodulin has been proposed as a ligand for the intracellular adaptor protein ezrin 81, a member of the ezrin‐radixin‐moesin (ERM) family of proteins that link transmembrane proteins to the actin cytoskeleton 82. This reinforces the likelihood of thrombomodulin‐mediating cell adhesion events. Consistent with this, knockdown of thrombomodulin can compromise the integrity of E‐cadherin‐mediated cell–cell contacts, potentially implicating thrombomodulin downregulation in the induction of EMT in cancer 43. A summary of thrombomodulin protein interactions is displayed in Fig. 3.

## CD248

CD248 also known as endosialin or tumour endothelial marker‐1 (TEM‐1) is the prototypical member of the CTLD group 14 family. It was first discovered as an antigen detected by the antibody FB5, which stained human tumour sections with patterns resembling blood vessels, but not healthy tissues 8. This led the authors to describe CD248 as a marker for tumour endothelium, although it could not be detected in cultured human umbilical vein endothelial cells (HUVEC). The study did however demonstrate that it was a highly glycosylated cell surface glycoprotein leading to its proposed name at the time; endosialin. CD248 was later identified as a marker of tumour endothelium in studies involving serial analysis of gene expression of vessels purified from human colorectal cancers in comparison to healthy colon vessels, hence its alternative name TEM‐1 83. Despite this, it is now widely accepted that CD248 is expressed by perivascular cells, stromal fibroblasts (especially in cancer and inflammation), mesenchymal stem cells and some tumour cells but not adult endothelium 84, 85, 86. The expression of CD248 on perivascular cells but not on endothelium *in vivo* was unequivocally demonstrated using multiple fluorescent labelling of human glioma sections 87. The study by St. Croix and colleagues which originally identified CD248 as TEM‐1 utilised CD146 or melanoma cell adhesion molecule (MCAM) antibodies to enrich the endothelium. Since MCAM also serves as a marker for pericytes, these samples likely contained perivascular cells as well as endothelium explaining the enrichment of CD248 84, 88. The proposed expression of CD248 on EPCs may have also added to this confusion 89.

## CD248 expression

CD248 is expressed during development and is first detected in mice at embryonic day 9.5 90. CD248 expression is mostly diminished in postnatal organs except for the kidney glomeruli and the uterus. Mice deficient in CD248 are viable and display no obvious defects, suggesting compensatory mechanisms may be employed during development 91. However, a marked decrease in tumour growth, metastasis and invasion was observed when CD248 deficient mice were challenged with human colorectal cancer xenografts. This defect in tumour growth and metastasis was only evident with abdominally implanted tumour cells, whereas subcutaneous implants displayed no difference relative to control animals. Further studies revealed that expression of CD248 exhibited negligible effects on primary tumour growth but increased metastasis formation in mouse models of breast cancer 92. Such prometastatic effects were attributed to CD248 expressing pericytes enhancing tumour cell intravasation. Elevated‐CD248 expression also correlated with greater metastasis and poorer survival in human breast cancer patients.

CD248 expression has been reported to be induced by hypoxia, predominantly involving the transcription factor hypoxia inducible factor‐2α (HIF‐2α) 93. This could explain the high levels of CD248 observed in the tumour microenvironment which is often poorly perfused and contains areas of hypoxia 94. Upregulation of CD248 can also arise in response to the growth factors FGF‐2, EGF and platelet‐derived growth factor‐BB (PDGF‐BB), which is further enhanced under hypoxic conditions 95.

CD248 expression has been described on naïve human CD8^+^ T‐cells, where it can negatively regulate proliferation 96. CD248 is expressed on stromal cells in secondary lymphoid organs and is required for correct secondary lymph node expansion in models of vaccination 97. However, CD248 was not essential for correct spatial organisation of T and B cells in this model.

## CD248 interaction partners and biology

CD248 has been reported to interact with the ECM proteins fibronectin and collagens I and IV 98. The interaction of CD248 with fibronectin increased cell adhesion of Chinese hamster ovary (CHO) cells overexpressing CD248 and was dependent upon the N terminus of fibronectin and the CTLD of CD248. Consistent with these data, the CTLD‐specific monoclonal antibody MORAb‐004 (ontuxizumab) could block CD248 binding to fibronectin and collagen I. Also, siRNA‐mediated knockdown of CD248 resulted in reduced migration and proliferation of fibroblasts, reinforcing a putative role in adhesion 99. Interestingly, a characteristic feature identified in CHO cells overexpressing CD248 is the upregulation of MMP9, thereby implicating CD248 in ECM degradation, a key step in sprouting angiogenesis as well as tumour metastasis and invasion 98. Further evidence in support of CD248 associating with the ECM stemmed from immunofluorescent staining with CD248 ECD fused to an Fc tag (CD248‐ECD‐Fc), this staining was only observed in the ECM from endothelial cells (HUVEC) and could partially co‐localise with fibronectin 100. More recently, we have shown direct interaction of CD248 with the endothelial ECM protein multimerin‐2 (MMRN2) 101. This interaction was dependent upon the CTLD of CD248 and CD248‐ECD‐Fc staining could partially co‐localise with MMRN2 on HUVEC; this may clarify previous findings involving the CD248‐ECD binding to the endothelial ECM 100.

Another ligand identified for CD248 was the secreted galectin‐3 (Mac‐2) binding protein Mac‐2BP and this interaction proved to be carbohydrate and Ca^2+^ independent 102. The CD248 interaction was mapped to two C‐terminal domains of Mac‐2BP and these have been previously implicated in binding galectin‐3, collagens V and VI, and nidogen, suggesting overlapping binding sites 103. This interaction invokes repulsion of human fibroblasts and HeLa cells expressing CD248 and Mac‐2BP, respectively. Moreover, this phenomenon was reduced following siRNA induced gene‐silencing of either molecule. Mac‐2BP is upregulated in the tumour cells of many different types of cancer and has been associated with increased metastasis and decreased survival in lung cancer patients 104. These findings strengthen the likelihood of CD248‐Mac‐2BP interactions occurring during tumorigenesis. It is currently unknown whether the therapeutic antibody ontuxizumab can block CD248 binding Mac‐2BP or MMRN2, a question that will likely impact novel future clinical interventions that target CD248.

**Figure 4:**
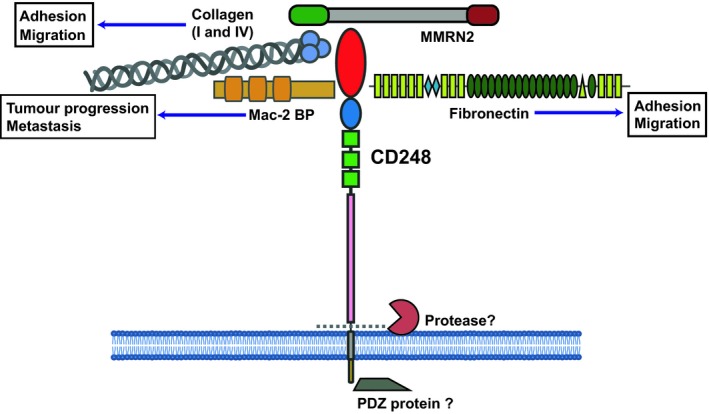
Schematic of CD248 structure with ligand binding partners. CD248 CTLD binds to fibronectin, Mac‐2 BP, Collagens I and IV and MMRN2. There are currently no known direct intracellular interaction partners for CD248.

There is evidence to suggest that the cytoplasmic tail of CD248 is involved in tumour development, as mice lacking this domain display reduced tumour growth in T241 fibrosarcomas and Lewis lung carcinomas (LLCs) 105. The cytoplasmic tail has also been predicted to contain a PDZ binding site and three potential phosphorylation sites, although to date identification of CD248 intracellular domain interactors have proved elusive 85, 106. A summary of CD248 protein interactions is summarised in Fig. 4.

## CD248 implications in angiogenesis

The role of CD248 in angiogenesis is complex and there is evidence to suggest it can both promote and inhibit angiogenesis depending on the circumstances. When CD248 is overexpressed in HeLa cells (normally lacking expression of the protein), multiple pro and antiangiogenic molecules are produced 95. CD248‐deficient mice displayed no gross defects in developmental angiogenesis or wound healing, but abnormalities were clearly apparent in tumour models of both the full gene deletion and the cytoplasmic deletion, resulting in smaller tumours exhibiting increased vessel density, although larger vessels were reduced 91, 105. Curiously, defects in tumour growth were not observed in all tumour models and the underlying mechanism remains unclear. Increased vascularity is also found in CD248‐deficient mouse models of glioblastoma multiforme, but there are no differences in tumour growth compared to wild‐type animals 107. These observations of increased vessel density may be rationalised by findings connecting CD248 with regulation of vascular patterning 87. This function of CD248 was uncovered when HUVEC treated with plate bound CD248 (to mimic pericyte expression), exhibited higher levels of apoptosis. This study highlighted the prospect of CD248 mediating a key role in vessel regression and pruning and emphasised for the first time that pericytes could be linked to such functions. Therefore, such defects in vascular pruning can result in an increase in microvessels that may have aberrant function. Moreover, these observations underline the possible therapeutic potential of the CD248‐ECD for inducing vessel regression and vascular normalisation, which might conceivably increase the delivery of chemotherapeutic agents into tumour tissue 108. Likewise, this vessel normalisation effect has been shown to allow more efficient infiltration of effector immune cells into tumours 109.

CD248 has also been implicated in the platelet‐derived growth factor (PDGF) signalling cascade 110. For example, following CD248 knockdown in pericytes, PDGF‐mediated proliferation is reduced *in vitro*. Furthermore, CD248 knockout mice displayed defects in sprouting angiogenesis but not splitting (intussusceptive) angiogenesis in skeletal muscle 111. Such defects could be recapitulated in mice treated with PDGFRβ inhibitors reinforcing a role for CD248 in PDGF signalling.

## CD248 in cancer

Elevated CD248 expression levels on tumour‐associated stroma have been reported in various primary tumour types including glioma, colorectal, melanoma as well as brain metastases 87, 112, 113, 114. CD248 expression has also been associated with worse outcome in patients with breast or colorectal cancer and could serve as a prognostic marker 115, 116. CD248 is expressed in numerous tumour cell lines and clinical samples of sarcomas and neuroblastomas, but is absent in cancer cells of epithelial origin 86. Indeed, highly malignant ‘side population’ sarcoma cells with some characteristics of cancer stem cells express CD248 117. These highly invasive side populations are also CD248^+^ in osteosarcoma 118. For these reasons, there has been a substantial drive into developing innovative strategies of targeting CD248 for tumour therapy.

Targeting CD248 has been attempted mainly by antibody‐based therapeutic approaches. One of the first preclinical attempts utilised single‐chain variable fragment (scFv) antibody‐like molecules generated against CD248 to successfully direct cytotoxic agents to neuroblastoma cells *in vitro* 119. Internalising antibodies against CD248 coupled with anti‐human IgG toxin‐conjugated antibodies revealed cell cytotoxic effects on CD248 expressing cancer cell lines *in vitro* 86. Such antibodies were developed as full antibody‐drug conjugate (ADC) molecules utilising conjugation to tubulin‐inhibiting drugs 120. Administration of these ADCs retarded tumour growth in multiple xenograft models. Another ADC against CD248 has been developed conjugated to a DNA‐binding duocarmycin derivative which has shown therapeutic efficacy in a human osteosarcoma xenograft model 121. The previously described CTLD‐specific CD248 antibody ontuxizumab has been utilised as a possible diagnostic imaging tool through use of ^125^Iodine conjugation and positron‐emission tomography 122. This technique resulted in rapid tumour uptake and real‐time visualisation of tumour burden and CD248 localisation in mice. Some more recent developments have involved the generation of human CD248 knock‐in mice to study the *in vivo* effects of ontuxizumab 123. Indeed, upon administration of ontuxizumab into B16 melanoma‐bearing mice, tumour growth was significantly reduced by up to 70%. This was presumably due to increase in microvessel density and the presence of nonfunctioning tumour blood vessels; phenocopying previous findings in CD248 knockout animals. This study also showed downregulation of surface expression of CD248 on pericytes by internalisation after ontuxizumab treatment *in vitro* and *in vivo*. Despite some preclinical efficacies, the ontuxizumab‐humanised CD248 antibody has recently completed two phase II clinical trials which failed to show improvements from standard of care or placebo in metastatic colorectal cancer and neglected to meet a progression‐free survival (PFS) goal of 35% in metastatic melanoma 124, 125 No clinical benefit was also observed in phase II trials involving metastatic soft tissue sarcoma treated with a combination with ontuxizumab and chemotherapy (gemcitabine and docetaxel) 126.

DNA vaccine approaches against CD248 have also been attempted preclinically, with antitumour effects being reported in both the prophylactic and therapeutic vaccine setting 127. The DNA construct consisted of murine CD248 fused to a fragment of tetanus toxoid, which circumvents tolerance to the self‐protein allowing triggering of an adaptive immune response. The authors described CD4^+^ and CD8^+^ T‐cell clones that were specific for CD248 epitopes as well as tumour specific antigens. The vaccination did not detrimentally affect wound healing or reproduction.

The targeting of CD248 may even be detrimental in some tumour types, as CD248 expression upregulated in hepatocellular carcinoma (HCC) patients in hepatic stellate cells (specialised pericytes found in the liver vasculature) was found to be protective correlating with better outcomes 128. Furthermore, inducible models of HCC in CD248 knockout mice displayed enhanced liver tumour progression relative to wild‐type controls.

## CD248 in inflammation and fibrosis

CD248 is expressed in mesenchymal stromal cells of developing mouse lymphoid tissues such as the spleen, thymus and lymph nodes 129, 130 expression in the adult mouse spleen is low, but is enhanced in stromal cells upon *Salmonella enterica* infection coinciding with spleen remodelling and repair 129. CD248 expression in mice is required for postnatal development of the thymus as well as regeneration of the thymus after *S. enterica* infection, in CD248 deficient animals this postinfection regeneration is impaired 130.

CD248 is expressed in vascular smooth muscle cells (VSMCs) undergoing proliferation and remodelling in apolipoprotein‐E (ApoE) KO mouse models of atherosclerosis as well as atherosclerotic lesions from patients 131. CD248 was shown to promote atherosclerosis as ApoE and CD248 double KO mice displayed less atherosclerosis when fed a Western style diet. These CD248‐deficient animals also exhibited marked reductions in macrophage infiltration into atherosclerotic plaques, due to reduced chemokine expression in VSMCs. Reduced macrophage recruitment in CD248 KO mice was also shown in other models of inflammation not involving atherosclerosis.

CD248 is expressed in the fibroblasts and pericytes of synovial tissue from patients with rheumatoid arthritis and psoriatic arthritis 132. CD248 expression has also been identified in the sublining layer of a distinct subset of synovial fibroblasts 133. CD248 knockout mice and mice lacking the cytoplasmic domain of CD248 both showed reductions in experimental arthritis compared to wild‐type animals, and displayed a marked reduction in synovial inflammation 132. CD248 also has roles in bone formation, it is expressed by bone forming osteoblasts but not bone removing osteoclasts in both mice and humans 134. CD248 knockout mice display denser bones most likely due to the hyperactivation of osteoblasts and increased mineral formation that would normally be inhibited by PDGF signalling, which is disrupted in CD248‐deficient cells. These findings suggest that targeting CD248 therapeutically may not only reduce inflammation in arthritis but also reduce bone loss associated with arthritis.

CD248 is expressed in healthy human and mouse kidney mainly in the mesangial cells of the glomerulus, but also in pericytes and fibroblasts. CD248 is upregulated in chronic kidney disease and inflamed kidney on a population of myofibroblasts and may be a useful predictor of renal failure 135. CD248 was later shown to have a potential role in the development of kidney fibrosis in mice 136, 137. CD248‐deficient mice undergoing renal damage were protected against fibrosis, this was not as a result of reduced inflammation but possibly due to CD248 KO fibroblasts producing less collagen and CD248 KO pericytes displaying impaired differentiation into myofibroblasts (a major cell type involved in fibrosis). Similar to fibrosis in kidney, CD248 deficiency also protected mice against liver fibrosis following liver injury, and these KO mice displayed reductions in collagen but no change in inflammation 138. CD248 expression was also detected in human samples of liver fibrosis on myofibroblasts and perivascular cells and was elevated in human liver fibrosis samples compared to healthy controls as well as correlating with levels of collagen deposition. Idiopathic pulmonary fibrosis patients also display high expression of CD248 in fibroblasts and it may serve as a disease severity marker 139.

CD248 has also been described as being highly expressed in skin samples of patients with systemic sclerosis in comparison to healthy controls 140. When CD248 expression was silenced by means of siRNA in mesenchymal stem cells from systemic sclerosis patients, TGF‐β and PDGF profibrotic signalling was reduced.

These described roles of CD248 promoting aberrant inflammation and fibrosis suggest it may be a suitable therapeutic target in certain diseases. However, it is possible that such CD248 inhibition may also have detrimental effects on lymphoid tissue remodelling and repair following infection.

## CD248 shedding

There are numerous reports describing soluble variants of CD248, suggesting its ECD may be shed from the cell surface as highlighted for other CTLD group 14 family proteins. CD248 has been suggested as a possible biomarker after it was purified from ascites fluid of patients with stage IV ovarian cancer 141, and pancreatic cancer 142. CD248 can be immuno‐precipitated from human serum in a fully glycosylated form of around 150–120 kDa, likely corresponding to the full ECD 143. In this same study, a highly sensitive and specific assay was developed using two different CD248 monoclonal antibodies to evaluate CD248 levels in patient blood. However, there was no significant difference in serum levels of soluble CD248 from colorectal cancer patients compared with healthy controls, which may limit its utility as a predictive biomarker particularly in this tumour setting. A protease capable of cleaving CD248 from the cell surface has yet to be identified.

## CD93

CD93 was first described as a receptor for the complement component C1q, hence its alternative name C1q receptor‐1 (C1qR1 or C1qRp) 144, 145. A subsequent study revealed that CD93 failed to engage C1q, but was instead implicated in cellular adhesion events 146. CD93 is expressed predominantly by endothelial cells and has been reported to be expressed by neurons in a rat model of inflammation and various cells of the haematopoietic system, including monocytes, neutrophils, B cells, natural killer (NK) cells, naïve T‐cells, platelets and haematopoietic stem cells 147, 148, 149, 150, 151, 152. It is also highly expressed on the tumour‐associated vasculature. In a recent example, elevated levels of CD93 expression were detected on human colorectal carcinoma sections 153. Interestingly, this study also examined soluble levels of CD93 within patient plasma and found a 30% reduction in colorectal carcinoma patients compared with healthy controls. CD93 has been described as a key gene in a proposed ‘tumour angiogenesis signature’ determined by meta‐analysis of 959 breast cancers, 170 renal cancers and 121 head and neck cancers 154. Moreover, CD93 has been identified as a member of a group of genes that are vastly upregulated in high‐grade glioblastoma tumour vasculature 155. This high expression profile was later confirmed at the protein level and correlated with poorer survival 156. Upregulated vascular expression of CD93 has also been described in nasopharyngeal carcinoma, as well as tumours of the eye including retinoblastoma and choroidal melanoma 157, 158, and correlates with a worse survival outcome. More recently, the CD93 CTLD has been derived from *Escherichia coli* expression systems that allow disulfide bond formation and has been purified to homogeneity allowing preliminary structural analyses using nuclear magnetic resonance approaches 159. This study revealed the CD93 CTLD does not bind Ca^2+^ and ongoing experiments will undoubtedly resolve the three‐dimensional structure and provide further molecular and functional insights into this family member.

## CD93 expression

During mouse development CD93 is expressed at embryonic day 9 and is detected in the vasculature including the inter‐segmental vessels 160. CD93‐deficient mice were viable and displayed no obvious abnormalities, but macrophages from deficient mice exhibited reductions in phagocytosis and reduced clearance of apoptotic cells in both *in vivo* and *in vitro* apoptotic clearance assays 161. A defect in antibody secretion in plasma cells was also a characteristic feature of CD93 knockout mice 152. Intriguingly, only CD93‐deficient female mice display aberrations in tumour growth and perfusion in orthotopic glioblastoma and fibrosarcoma models 156.

## CD93 interaction partners and biology

Silencing of CD93 by RNA interference in HUVEC‐impaired proliferation, migration, adhesion and sprout formation 162. Subsequent studies validated these effects with disruptions observed in adhesion, migration and tube formation 156, 157. A monoclonal antibody raised against human CD93 (clone 4E1) which binds between the CTLD and sushi domains demonstrated antiangiogenic activity in Matrigel assays both *in vitro* and *in vivo,* reiterating its roles in endothelial biology 162. CD93 has been identified as a gene that is downregulated upon VEGF blockade by using bevacizumab in patented studies performed by Genentech 163. Similarly, another report highlighted that CD93 protein expression was diminished upon pharmacological inhibition of VEGFR2 and fibroblast growth factor‐1 with brivanib alaninate 164. While VEGF could be having effects on CD93 independent of angiogenesis, the loss of function experiments involving CD93 seem to steer towards a proangiogenic function. Ligand binding studies of CD93 with a variety of ECM proteins revealed a lack of binding to all proteins tested *in vitro* including; collagen I and IV, gelatin, laminin, vitronectin and fibronectin 165. The only known extracellular interacting partner for CD93 was recently identified as the endothelial specific ECM protein MMRN2 101. This interaction is dependent on the CTLD of CD93 and by combining structural modelling with site‐directed mutagenesis a predicted long‐loop region of this structure was proposed to be critical for binding to MMRN2. This offers a platform for developing innovative therapeutics that specifically target CD93 to interrupt this interaction. The CD93‐MMRN2 interaction was later independently validated and surface plasmon resonance was used to characterise the interaction and determine binding affinities 166. A key residue within the coiled‐coil domain of MMRN2 (F238) was proposed as being integral for CD93 binding. Interestingly, this study also provided an explanation for the previously described antiangiogenic effects of the CD93 antibody 4E1, as it could interrupt the CD93‐MMRN2 interaction.

The CD93‐MMRN2 interaction was also shown to be involved in the proper deposition and organisation of fibronectin a process termed fibrogenesis 167. In CD93‐deficient mice the fibronectin matrix was disrupted in vessels of postnatal retinas and vessels in orthotopic models of glioblastoma 167. In the same study, the use of specific antibodies that detect activated α5β1 integrins revealed disruption of this activated integrin in CD93 knockout mice. During postnatal retinal angiogenesis, CD93 is expressed on filopodia while MMRN2 expression is absent from these protrusions but present in the surrounding ECM. Finally the authors showed that MMRN2 and fibronectin expressions are upregulated in high grade human glioma 167. Co‐localisation of CD93 and MMRN2 expressions has been demonstrated in vessels of a range of different solid human tumours including melanoma, Ewing's sarcoma, ovarian carcinoma and glioma amongst others 166, 167. There are likely other partner proteins for CD93 as a study showed that the recombinant form of the CD93 ECD can engage the cell surface of THP‐1 cells indicating the expression of a currently unknown CD93 ligand in this monocyte cell line 168.

**Figure 5:**
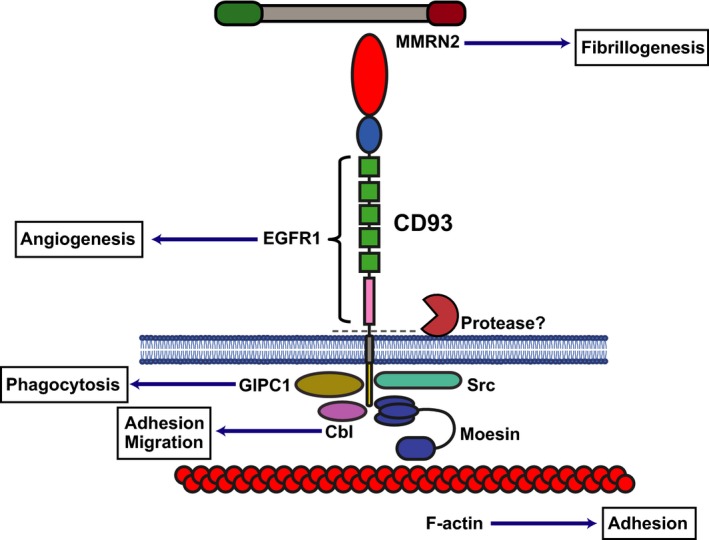
Schematic of CD93 structure with ligand binding partners. CD93 CTLD binds to MMRN2. The whole ECD has been shown to be cleaved by an as yet unidentified metalloproteinase. The intracellular cytoplasmic domain binds to moesin which in turn links CD93 to the actin cytoskeleton. The cytoplasmic domain also binds to Cbl and GIPC1 and src.

The cytoplasmic domain of CD93 encompasses a positively charged juxtamembrane region that binds to the adaptor protein moesin 169. Moesin is a member of the ERM family of proteins, which like ezrin and radixin, anchor proteins to the actin cytoskeleton 82. In knockdown studies involving CD93, adherens junctions were disrupted 156. Strikingly, reintroduction of wild‐type CD93 but not CD93 lacking the moesin binding motif, restored adhesion junctions and highlighted the importance of CD93‐moesin interactions in maintaining the integrity of endothelial cell adhesion *in vitro*. Relatedly, CD93‐deficient mice display increased permeability in blood vessels possibly due to disruptions in tight junctions 170. Another intracellular binding partner for CD93 has been defined as GIPC, (Gα interacting protein (GAIP)‐interacting protein C‐terminus), 171 an adapter protein that contributes to arterial maturation and mural cell coverage 172. The binding of GIPC was dependent on the positively charged juxtamembrane as well as the final C‐terminal 11 amino acids of the cytoplasmic tail. CD93 was originally predicted to bind to the E3 ubiquitin ligase Cbl, due to the CD93 cytoplasmic domain containing a binding motif that is also found in the Cbl binding protein APS (adapter with pleckstrin homology and Src homology‐2 domains) 173. CD93 binding to Cbl was proved experimentally by co‐immunoprecipitation in HUVEC, and this interaction was abolished upon knockdown of the ECM adhesion molecule β‐dystroglycan 165. This study proposed that the cross‐talk between the laminin‐binding protein β‐dystroglycan and CD93 led to endothelial cell adhesion and migration. The authors suggested that upon laminin binding to β‐dystroglycan src kinase phosphorylates specific tyrosine residues in the cytoplasmic tail of CD93, which in turn facilitates binding to Cbl. In this setting, Cbl may serve as an adapter protein rather than a ubiquitin ligase. A summary of the protein interaction partners of CD93 are summarised in Fig. 5.

## CD93 and inflammation

Mice deficient in CD93 when subjected to experimental peritonitis displayed increased leucocyte infiltration, and this effect was not restricted to a particular cell type 170. CD93 also has been suggested to have neuroprotective roles and as it is upregulated in murine models of stroke 174. This upregulation effect was also observed at the protein level in several cell types including endothelial cells, microglia and macrophages. Moreover, cerebral ischaemia in CD93 knockout mice resulted in enhanced neuro‐inflammation compared to wild‐type animals. CD93 knockout mice also displayed increased brain and spinal cord inflammation when compared to wild‐type mice in two different models of encephalomyelitis 175. Based on the CD93 expression profile within the tumour vasculature and its potential anti‐inflammatory roles, it is plausible to contemplate that CD93 may serve as an immunosuppressive molecule in the tumour microenvironment, limiting immune cell infiltration and facilitating tumour immune evasion mechanisms.

## CD93 shedding

Soluble CD93 has been detected in human plasma, described to be a protein released from HUVEC and also a component of their ECM 171, 176, 177. Several studies have highlighted that levels of soluble CD93 directly correlate with disease status; in plasma it has been proposed as a potential biomarker for coronary artery disease and is elevated in synovial fluid of rheumatoid arthritis patients 168, 178. In another study, soluble CD93 was proposed as a marker for inflammation as it is reportedly shed from the cell surface of monocytes and neutrophils, which is likely to be dependent on metalloproteinases, although the major sheddase ADAM17 is not involved 179. This cleavage event most likely liberates the entire ECD of CD93 and can be stimulated by TNFα or LPS. This inflammation induced shedding of CD93 was subsequently confirmed by *in vivo* experiments, and macrophages were suggested as the main source of soluble CD93 180. Conversely, elevated levels of soluble CD93 in peritonitis fluid were shown to be dependent on nonhaematopoietic cells, likely from endothelium 170. Soluble CD93 has been suggested to induce differentiation of monocytes by as yet undefined mechanisms 168. A role for soluble CD93 in the process of efferocytosis has been proposed, whereby cleaved CD93 binds to apoptotic cells via the CTLD in a Ca^2+^ independent manner 181. Soluble CD93 then acts as an opsonin‐coating apoptotic cells and is in turn bound by α_x_β_2_ integrin on macrophages via the CD93 EGF repeats aiding phagocytosis.

Soluble recombinant CD93 encompassing solely the five tandem EGF repeats and mucin domain of CD93 mediate proangiogenic effects on endothelial cells, increasing proliferation and migration of HUVEC and promoting angiogenesis *in vivo* 182. Although these proangiogenic signals were also induced with the full‐length CD93‐ECD, constructs lacking the CTLD elicited more potent effects by enhancing the EGFR1 mediated PI3K signalling pathway; similar results were observed with recombinant soluble forms of thrombomodulin encompassing the EGF and mucin domains 36.

The O‐glycosylation modifications within the mucin‐like domain of CD93 contribute to stabilising its cell surface expression 183. Intriguingly, the lack of O‐linked glycosylation enhanced proteolytic cleavage of CD93 from the cell membrane and increased levels in culture medium. This provides a possible role for the mucin‐like region within all the CTLD group 14 family members in preventing proteolytic cleavage, and also offers a potential mechanism of modulating surface cleavage events. The cell‐surface expression of CD93 is regulated by protein kinase C isoenzymes 184, and shedding could be enhanced by phorbol 12‐myristate 13‐acetate (PMA), a potent activator of protein kinase C 185.

Upon knockdown of MMRN2, together with inhibition of new protein synthesis by cycloheximide treatment, cell surface CD93 levels were shown to be diminished whereas soluble CD93 levels increased 167. This suggests that the interaction with MMRN2 may render CD93 less susceptible to proteolytic cleavage and hence this recognition event is important for regulating stable cell surface expression of CD93.

## Additional roles for CD93

A study examining CD93 expression in neurons and microglia revealed that upon response to LPS mediated inflammation, the cytoplasmic tail of CD93 could be detected in the cytoplasm and nucleus 148. This is the first instance that a possible gene expression modulating role has been inferred for the CD93 cytoplasmic region. As CD93 ECD cleavage is enhanced by LPS, and the cytoplasmic domain can be detected even after cleavage 171, it is possible that it translocates to the nucleus after ECD shedding, similar to that described for notch ECD 186. The authors did not confirm whether nuclear localisation followed CD93 ECD cleavage and further work is warranted in order to define the precise molecular mechanisms underlying this effect. Notably, similarities have been proposed between CD44 and CD93 171 as the CD44 ECD can be cleaved by ADAM10 and its intracellular domain by γ‐secretase, similar to that described for notch receptors 187.

## CLEC14A

C‐type lectin family 14 member A (CLEC14A) is a type‐I single‐pass transmembrane glycoprotein and considered to be endothelial specific. It was described as a novel endothelial‐specific gene identified by microarray analysis and data mining, and referred to as an unidentified expression sequence tag (EST; accession number http://www.ncbi.nlm.nih.gov/protein/AW770514) 188. CLEC14A was initially classified as a tumour endothelial marker based on immunohistochemical staining of multiple distinct tumour types, with strong staining on tumour‐associated vessels in contrast to a near absence of staining in healthy tissues 189. Upregulation of CLEC14A at the mRNA level was also described in nonsmall cell lung cancer (NSCLC) tissues compared to healthy lung 190. Interestingly, high expression of CLEC14A in this cancer type correlated with improved clinical outcomes. A further study indicated that the methylation status of *CLEC14A* strongly correlated with its expression levels in NSCLC, and CLEC14A protein levels were reduced in tumour tissues compared to healthy adjacent tissue 191. Similar to CD93, CLEC14A was described as a key gene in a proposed ‘tumour angiogenesis signature’ determined by meta‐analysis of over 1000 tumour samples including breast, renal and head and neck cancers 154. It was subsequently found to be upregulated at the protein level and increased with tumour progression in two different spontaneous mouse tumour models, namely cervical and pancreatic 177. More recently, CLEC14A overexpression on the vasculature in ovarian cancer has been reported but did not correlate with survival in this tumour type 192. The authors also demonstrated that CLEC14A expression was undetectable along with reductions in microvessel density in patients receiving neoadjuvant chemotherapy prior to surgery.

## CLEC14A expression

CLEC14A (or C1qrl in zebrafish) is thought to be located downstream of the master endothelial and haematopoietic regulatory transcription factor etsrp in zebrafish (ETV2 in humans) 193. The etsrp transcription factor has recently been implicated in tumour angiogenesis in xenograft models of melanoma and sarcoma in zebrafish embryos 194. During zebrafish development, clec14a is expressed at 24 h postfertilisation and morpholino knockdown of gene expression can have detrimental effects on vasculature formation 189. Interestingly, following reintroduction of human CLEC14A mRNA into these knockdown zebrafish embryos, the vasculature reverted back to a normal phenotype showing the correct zebrafish homologue was targeted and highlighting the conserved nature of these genes. Zebrafish homozygous for clec14a null alleles develop normally into viable adults 195, these zebrafish as embryos do however display delays in vasculogenesis and angiogenesis, and these defects can be heightened with knockdown of C1qr/CD93.

Mouse embryos display expression of CLEC14A in inter‐segmental vessels and vessels in the developing brain, at embryonic day 10.5 196. Expression was also detected in the vessels of mouse retinas at postnatal day 12, which are constantly undergoing development after birth. CLEC14A has also been described as being upregulated when EPCs differentiate into outgrowth endothelial cells 197. Despite early expression in embryogenesis, CLEC14A is not completely critical to angiogenesis as CLEC14A deficient animals develop normally with no gross defects in vessel formation.

CLEC14A has been described to be upregulated by low shear stress 189. Indeed, upon application of 2 pascal (Pa) of flow induced laminar shear stress to HUVEC in culture, this leads to a significant reduction (> 90%) of CLEC14A expression when compared to static culture (low shear stress). This may explain the expression of CLEC14A observed within the ill‐formed vessels of tumours that experience irregular blood flow and low shear stress 198. Upstream regions of the CLEC14A gene in humans contain predicted Sp1 transcription factor binding sites. Interestingly, Sp1 is phosphorylated in response to shear stress and can inhibit the expression of membrane type‐I matrix metalloproteinase (MT1‐MMP) in endothelium 199. Microarray analysis of atherosclerosis patient samples revealed upregulation of CLEC14A in vessels that display high levels of stenosis 200. This is consistent with previous findings, as shear stress is lower in blood vessels containing atherosclerotic plaques when compared with healthy controls 201. CLEC14A expression has also been linked with hypoxia in HUVEC, and could explain its greater expression in the tumour vasculature 202.

CLEC14A expression has been demonstrated in two different human lung cancer cell lines *in vitro* and when CLEC14A was further overexpressed in these cell lines, this led to reductions in proliferation, migration and invasion as well as reductions of *in vivo* tumour formation as xenografts in nude mice 191. However, the physiological relevance of CLEC14A expression in tumour cells themselves remains to be seen as such expression has not been reported in clinical specimens or in tumour cells by any other group.

## CLEC14A roles in vascular biology and cancer

The involvement of CLEC14A in angiogenesis is reinforced by *in vivo* experiments performed in homozygous CLEC14A knockout mice 203. These mice remained viable and displayed no gross developmental defects. Nevertheless, when challenged with subcutaneous LLC, tumour growth and tumour angiogenesis were reduced relative to wild‐type controls. Similarly, in subcutaneous sponge implants FGF‐2‐induced angiogenesis was also impaired. However, another report has suggested that CLEC14A may not serve as a viable antivascular target; this study demonstrated that although implanted tumour growth of LLC and B16F10 melanoma was markedly impaired in CLEC14A knockout mice in comparison to wild‐type littermates, tumour bearing CLEC14A knockout mice died earlier 204. These deleterious effects were attributed to reduced pericyte coverage and CLEC14A‐deficient vessels displaying increased permeability. Furthermore, this study revealed that CLEC14A deficiency led to increased lung metastasis burden when B16F10 cells were injected intravenously or into the foot pad.

There are conflicting results regarding vessel sprouting from aortic ring assays from CLEC14A KO mice. Noy *et al*. described reduced sprouting in these CLEC14A‐deficient mice, but Lee *et al*. described increased sprouting in aortic ring assays in comparison to control 203, 204. The reasons for these described conflicting roles for CLEC14A in angiogenesis have not been elucidated. It is possible that discrepancies are due to differences in methodology of these assays or dissimilarities in background strains of these CLEC14A KO mice.

The requirement for CLEC14A in various *in vitro* angiogenesis assays were reported by two independent groups utilising siRNA‐mediated knockdown of *CLEC14A* 189, 196. Based on these knockdown experiments, the ability of HUVEC to form tubes and close wounded monolayers in scratch assays was compromised. In addition, involvement of CLEC14A in sprouting angiogenesis was demonstrated by siRNA knockdown of *CLEC14A* in HUVEC which led to marked reduction in sprout formation based on spheroid assays, *CLEC14A* deficient cells were also less likely to be found as tip cells in these sprouts 203. Ectopic expression of CLEC14A in cells that do not normally express it, results in the formation of filopodia‐like protrusions 189. Altogether these findings implicate CLEC14A in filopodia formation, a vital step in sprouting angiogenesis.

The CLEC14A CTLD has been implicated in cell–cell adhesion interactions, since CLEC14A overexpressing HEK293F cells have the ability to initiate preliminary cell–cell aggregates, which can be abolished following incubation with CTLD‐specific CLEC14A antibodies 205. These antibodies were reactive against both human and mouse CLEC14A forms and could downregulate CLEC14A levels on the surface of HUVEC, posing a potential for internalisation of antibodies, and possible utilisation as ADCs carrying a cytotoxic payload. Finally, these antibodies could reduce HUVEC cell migration and tube formation based on *in vitro* assays. Further studies optimised the solubility and stability of the CLEC14A CTLD‐targeting antibodies and showed that they could block angiogenesis in mouse models utilising Matrigel plugs injected with recombinant VEGF or human tumour cells 206. Collectively, these results suggest that the CTLD of CLEC14A has functional roles in angiogenesis.

## CLEC14A interaction partners

CLEC14A has been described as a component of HUVEC ECM which binds to the ECM glycoprotein MMRN2 177. Like CLEC14A, MMRN2 protein was upregulated with tumour progression of two different spontaneous mouse cancer models, highlighting importance of this interaction and potential as therapeutic tumour vascular targets 177. The CLEC14A–MMRN2 interaction could be blocked by a monoclonal antibody specific for CLEC14A, and when administered intraperitoneally retarded growth of subcutaneously implanted LLC in mice 203. This interaction was dependent upon a predicted long‐loop region encompassing residues 97–108 within the CLEC14A CTLD 101. The CLEC14A‐MMRN2 interaction could also be targeted using a minimal peptide fragment derived from MMRN2. This peptide reduced endothelial tube formation and also decreased tumour growth when expressed by LLC cells *in vivo* 101.

The CLEC14A CTLD also has the capacity to bind other ligands including the heat shock protein 70 kDa 1A (HSP70‐1A) which increased HUVEC adhesion, aggregation and ERK phosphorylation 207. This finding may rationalise the cell aggregation effects observed in HEK293F cells overexpressing CLEC14A, with HSP70‐1A forming oligomeric complexes and creating a bridge between CLEC14A expressed on different cells. This binding phenomenon was dependent on amino acids 43–69 of the CLEC14A CTLD 207, which based on its predicted structure encompasses an alpha helical region that is distal to the MMRN2 binding site. However, at present, it is unclear whether HSP70‐1A and MMRN2 are mutually exclusive binding events or if they compete with each other 101. The same group previously discovered that HSP70‐1A could serve as a potent proangiogenic factor 208. The active HSP70‐1A binding region of CLEC14A fused to an Fc tag was used to create a novel peptibody which could inhibit HSP70‐1A–stimulated tubule formation of HUVEC *in vitro* 207. In the same study, stimulation of HUVEC with HSP70‐1A increased ERK phosphorylation, and this effect was reduced when incubating with CLEC14A CTLD‐Fc fusion proteins. This suggests that CLEC14A may have signalling roles, although the authors did not probe whether HSP70‐1A–mediated ERK phosphorylation was blocked with knockdown of CLEC14A.

**Figure 6:**
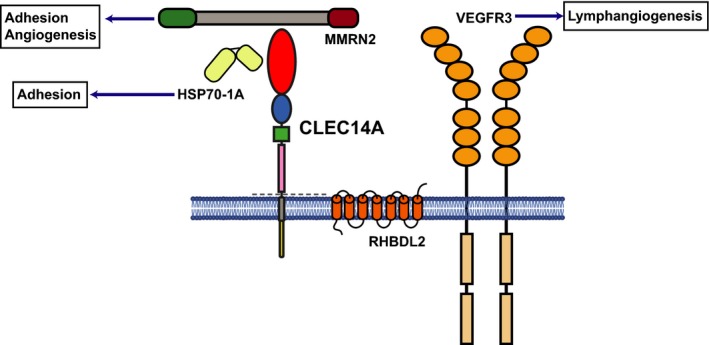
Schematic of CLEC14A protein with ligand binding partners. CLEC14A CTLD binds to MMRN2 and to HSP70‐1A. The whole ECD can be cleaved by RHBDL2. There are currently no known direct intracellular partners for CLEC14A.

The intracellular domain of CLEC14A reportedly interacts with vascular endothelial growth factor receptor‐3 (VEGFR‐3) 204. There are currently no other known interactors for the CLEC14A cytoplasmic domain, although global phosphoproteomic analysis of HUVEC has revealed the presence of five serine residues that can be phosphorylated, namely S437, S445, S483 S487 and S488 209, 210. The phosphorylated S483 was also found in other proteomic analyses and was described as being close to a predicted PDZ binding domain in the CLEC14A cytoplasmic domain 177. Since these residues are not conserved in mouse CLEC14A the relevance of these post‐translational modifications will need to be determined experimentally. A summary of the protein interactions of CLEC14A are shown in Fig. 6.

## CLEC14A shedding

CLEC14A can be shed from the endothelial cell membrane by the intramembrane serine protease RHBDL2 211. RHBDL2 cleaves at a site close to the transmembrane domain, liberating the intact ECD of CLEC14A to regulate sprouting angiogenesis. The CLEC14A‐ECD can mediate antiangiogenic effects *in vitro* and *in vivo* when utilised as an Fc‐tagged recombinant protein. Intriguingly, when used as a staining reagent the CLEC14A‐ECD‐Fc fusion bound to sprouting endothelial cells, predominantly tip cells. Therefore, one could propose a scenario in which shedding of CLEC14A may aid in regulation of sprouting angiogenesis. Such cleaved CLEC14A predominantly by stalk cells in an angiogenic sprout would then be able to bind to tip cells.

## CTLD group 14 family summary

The CTLD group 14 family members all mediate effects upon the vasculature and some share remarkable similarities with respect to binding partners localised within the extracellular matrix. Further similarities are also observed with respect to expression patterns and regulation by predicted transcription factors. A summary of similarities and differences are displayed in Table 1.

**Table 1:** Biological comparisons of CTLD group 14 family members.

Biology	Thrombomodulin	CD248	CD93	CLEC14A	References
Knockout mouse	Embryonic lethal	Viable	Viable	Viable	15, 91, 161, 203
Knockout mouse tumour development	N/A	Reduced growth	Reduced growth	Reduced growth	91, 156, 203
Extracellular binding partners	Thrombin, Protein C, Lewis Y antigen, FGFR1, Fibronectin, GPR15 (EGF5), Ang‐1, Ang‐2, CD14, HSP70‐1	Mac‐2BP, Fibronectin, Collagens I & IV, MMRN2	EGFR1 (EGF domains), MMRN2, αxβ2	MMRN2, HSP70‐1A	19, 24, 25, 31, 34, 37, 38, 59, 60, 98, 101, 102, 177, 181, 182, 207
Intracellular binding partners	Ezrin	None reported	Moesin, GIPC, Cbl, src	VEGFR3	81, 148, 165, 169, 171, 204
Expression	Endothelial, Haematopoietic	Pericytes, Fibroblasts, CD8+ T cells	Endothelial, Haematopoietic, Neural	Endothelial	12, 14, 27, 84, 95, 96, 148, 156, 189, 196
Shear‐induced expression	Downregulated with shear	Not reported	Not reported	Downregulated with shear	57, 189
Cleavage	Whole ECD, Possibly CTLD	Not reported	Whole ECD	Whole ECD	70, 77, 179, 211
Cleavage enzyme	RHBDL2, MMPs neutrophil elastase, cathepsin G, proteinase 3	Not reported	Metalloproteinases	RHBDL2	70, 72, 179, 211
Location of soluble form	Culture medium, Blood, Urine, Synovial fluid	Blood, Ascites	Culture medium, Blood, Synovial fluid	Culture medium, Urine	30, 55, 73, 74, 75, 76, 141, 143, 153, 158, 178, 179, 180, 211, 228
Solved structures	EGF domains in complex with thrombin	Not reported	Not reported	Not reported	20

## Expression localisation of CTLD group 14 family members *in vivo*

**Figure 7:**
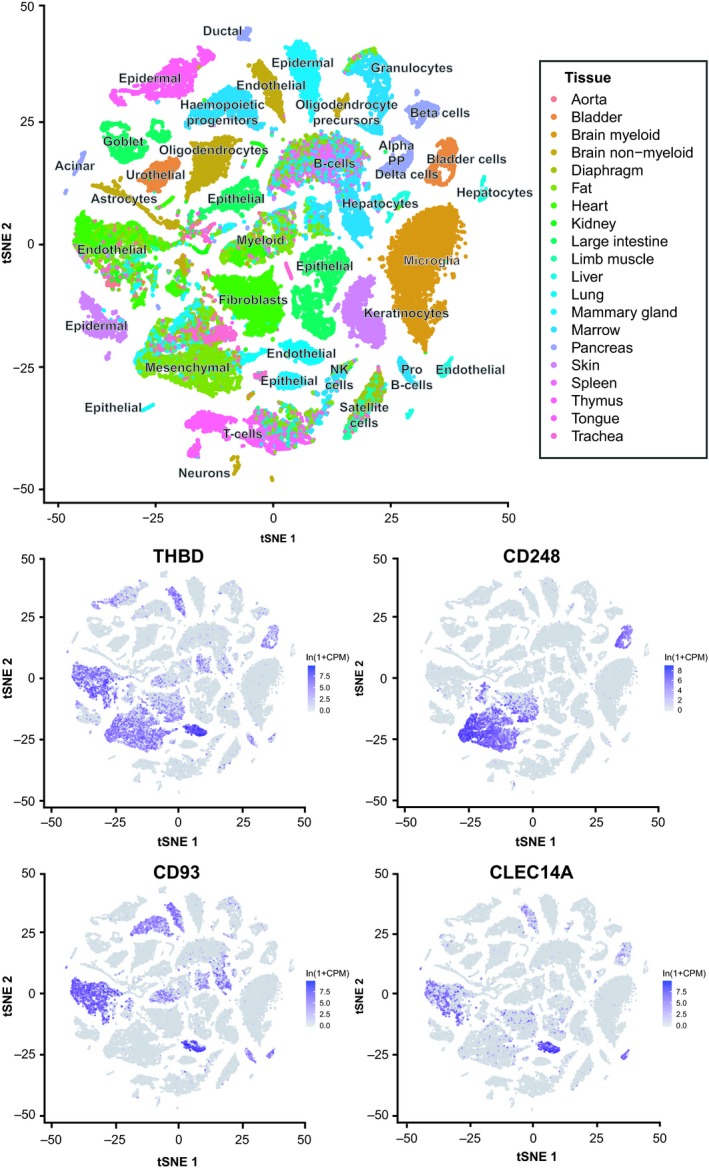
Expression of CTLD group 14 family members in mouse tissues. The Tabula Muris database was used to determine which mouse cell types expressed each CTLD group 14 family gene from data acquired through fluorescence activated cell sorting and single‐cell gene expression analysis. The t‐SNE plot at the top displays annotations of each cell type and shows a legend of colours corresponding to which organ or tissue type that cell was from. The lower t‐SNE plots display in which cell types each family member was expressed (purple), ln(1 + CPM) is the natural logarithm of counts per million + 1.

**Figure 8:**
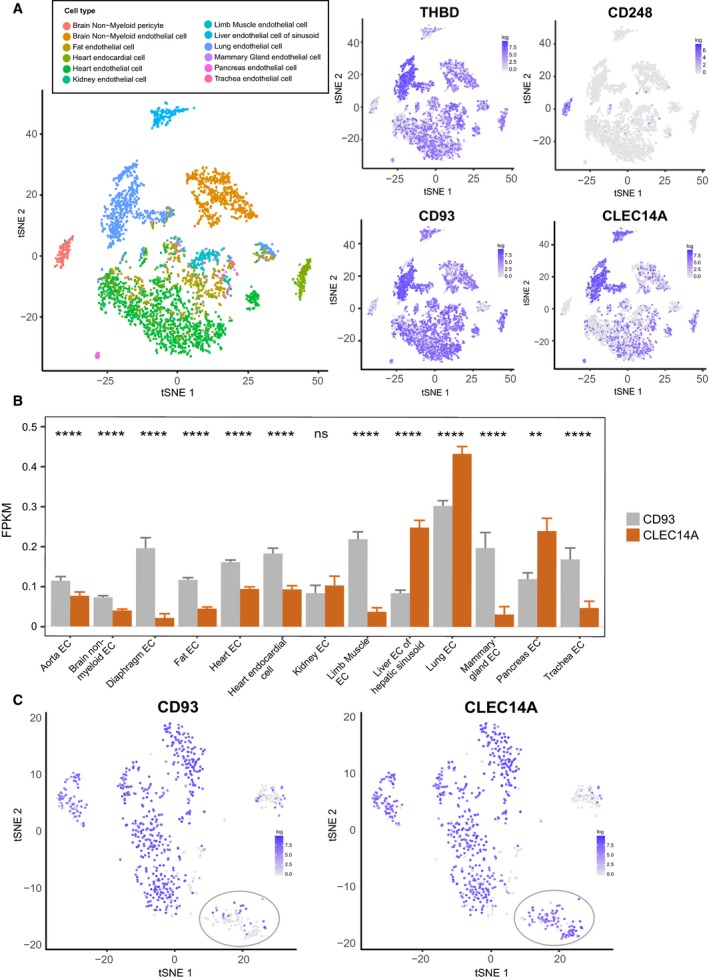
Endothelial expression of CTLD group 14 family members in mouse tissues. (A) The Tabula Muris database was used to create t‐SNE plots of all endothelial cells from different organs as well as brain pericytes. The t‐SNE plot at the top left displays a legend of colours corresponding to which organ or tissue type that cell was from. Expression of each CTLD group 14 family member within these cell types are displayed as t‐SNE plots. (B) Single‐cell sequencing data analysed as fragments per kilobase million was used to compare CD93 and CLEC14A expression in different endothelial cells from different organs. Wilcoxon statistical test was used to compare ****P ≤ 0.0001 Aorta ECs *n* = 262, Brain nonmyeloid EC *n* = 1250, Diaphragm EC *n* = 154, Fat EC *n* = 1180, Heart EC *n* = 2274, Heart endocardial cell *n* = 350, Kidney EC *n* = 80, Limb Muscle EC *n* = 258, Liver EC *n* = 392, Lung EC *n* = 1476, Mammary gland EC *n* = 98, Pancreas EC *n* = 98, Trachea EC *n* = 66. (C) t‐SNE plots of lung endothelium alone were created which revealed the presence of a cluster of cells expressing low levels of CD93 when compared with all other lung endothelial cells but similar levels of CLEC14A (grey ellipse).

To gain an in‐depth understanding of gene expression of all four CTLD group 14 family members *in vivo* we used the recently described Tabula Muris database, which consists of single‐cell transcriptomic analyses of over 100 000 cells derived from 20 different healthy adult mouse organs and tissues from C57BL/6 strain mice 212. This allowed graphical representation of gene expression by use of t‐SNE plots and revealed that thrombomodulin is mainly expressed in endothelium, epithelium and mesenchymal cell types, as well as some myeloid, pro‐B cell and bladder cells (Fig. 7). CD248 is restricted mainly to mesenchymal cells, fibroblasts, pericytes and bladder cells, and importantly there was a lack of expression of CD248 in endothelial cells from multiple organs. CD93 showed mainly endothelial, as well as myeloid, pro‐B cell and haematopoietic progenitor cell expression, but a lack of expression in neurons. Finally, CLEC14A exhibited the most endothelial specific expression of the four family members but also localised in bladder cells and leucocytes from the thymus. Bladder cells described here include mesenchymal cell types. The endothelial expression of CTLD group 14 family members was then investigated further, t‐SNE plots of all endothelial cells from different organs as well as pericytes from brain were created, showing that thrombomodulin is expressed in mostly all endothelial cell types, CD248 is not expressed in endothelial cells (but is expressed in pericytes) and CD93 and CLEC14A are expressed to a varied degree in most endothelial cells (Fig. 8A). As CD93 and CLEC14A share the ligand MMRN2, they are both expressed by endothelium, share similar endothelial phenotypes and have been suggested previously to compensate for lack of the other, we investigated whether endothelial cells in certain organs displayed differential expression of each gene. This revealed that CD93 is expressed higher than CLEC14A in a majority of organs except kidney (no significant difference) and liver, lung and pancreas endothelium, where CLEC14A is expressed significantly higher (Fig. 8B). Interestingly, there appeared to be a subset of endothelial cells within the lung that do not express CD93 but do express CLEC14A, t‐SNE plots solely of lung endothelium showed that there was a clustering of these cells suggesting an unknown endothelial subtype that does not express CD93 in mouse lung (Fig. 8C). The Tabula Muris database provides novel interesting insights into expression patterns, at least at the gene expression level, in an adult healthy mouse, although this is not an exhaustive list of all mouse cell types that express these genes as only 20 major organs and tissues were analysed. Similar studies analysing single‐cell gene expression of mice in different disease states such as cancer or inflammation would be an extremely valuable resource.

## Considerations and perspectives

### Similarities between CTLD group 14 family members

All CTLD group 14 family members comprise six canonical cysteines in the CTLD that are predicted to form disulfide bonds and support the CTLD scaffold. They also encompass two noncanonical cysteines located within the predicted long‐loop regions which due to their close proximity may also form disulfide links. Interestingly, such noncanonical cysteines within the long‐loop region are found only in three other CTLD families; group 8 containing layilin and chondrolectin, groups 11 and 12. Disulfide bond formation within this long loop appears to be essential for the interaction of CLEC14A and CD93 with MMRN2. Upon point mutation of these long‐loop cysteines, the CLEC14A CTLD folds correctly as it is recognised by conformation‐specific monoclonal antibodies, but completely diminishes its binding capability with MMRN2 101. It is possible that the corresponding cysteines in CD248 and thrombomodulin are similarly important for CTLD‐mediated recognition events and constructs containing point mutations of these residues could represent invaluable tools in determining functional relevance of the CTLD within this family.

Thrombomodulin and CD93 are both anchored to the actin cytoskeleton by associating with ERM adapter proteins; thrombomodulin to ezrin and CD93 to moesin. The thrombomodulin–ezrin interaction was initially described in epithelial cells and this is not surprising given that ezrin in this cell type is the highest expressed ERM protein. In contrast in endothelial cells, moesin is the most abundantly expressed ERM protein 213. Due to the high sequence homology between ezrin and moesin (~ 75% sequence identity) 214, it is tempting to speculate that thrombomodulin also interacts with moesin in the endothelium. However, the ability of thrombomodulin to bind to ezrin or moesin within endothelial cells is yet to be assessed. Likewise, CD93 may be able to bind to multiple ERM adapter proteins as is the case for CD44 binding to ezrin, radixin and moesin 215. Both thrombomodulin and CD93 interactions with ERM adapter proteins are dependent upon positively charged residues within the cytoplasmic tail, which are absent in the corresponding regions of CD248 and CLEC14A. This motif comprises of RKK in thrombomodulin and RKR in CD93. Strikingly, the RKE motif in CLEC14A could potentially abolish binding to ERM proteins due to repulsion effects attributable to the negatively charged glutamic acid side chain. Nevertheless, there is a distinct possibility that the intracellular domain of CLEC14A makes direct or indirect contacts with the cytoskeleton, due to its proposed roles in filopodia formation and cell migration. Also, the corresponding region in the CD248 intracellular domain consists of the NKR motif, and it is unclear whether the noncharged asparagine residue can compensate for binding to ERM adapter proteins. Finally, a related point to consider is that in both CD248 and CLEC14A, the three‐amino acid motif is preceded by a proline residue which may cause rigidity and/or conformational alterations that could affect interactions with ERM adapter proteins.

Evidence for CLEC14A along with thrombomodulin acting as potential cell adhesion molecules is observed following overexpression of each protein and leads to induced cell aggregation. Such effects are dependent upon the CTLD of each protein 80, 196, 205. HUVEC plated on immobilised fragments of MMRN2 are sufficient to allow adherence of HUVEC in cell binding assays; however, at present, it is unclear whether CLEC14A, CD93 or both mediate this adhesive function 101. Similarly, when CD248 is overexpressed in CHO cells, this enables them to bind to fibronectin and Matrigel in cell adhesion assays 98.

CLEC14A and CD93 both bind MMRN2 as does CD248, this to our knowledge is the first example of an endothelial protein binding to an extracellular matrix protein which in turn interacts with a pericyte‐expressed protein of the same family. This raises an interesting question of how MMRN2 has evolved to bind two distinct CTLD group 14 family members in nonoverlapping regions of the same molecule. This binding event may have roles in already proposed CD248‐dependent vascular regression caused by pericytes 87. With regard to CLEC14A expression and MMRN2 interaction, this may flag areas of the newly formed vasculature that is experiencing low blood flow and low shear stress. Upon binding to MMRN2 through CD248, pericytes could then selectively cause vascular regression through unknown mechanisms. Interestingly, pericyte coverage of endothelium is reduced in the brain, retina and melanoma tumour vasculature of CLEC14A knockout mice 204. However, no defects were reported in pericyte coverage of vessels in models of gliomas between CD93 knockout and wild‐type mice 156. This suggests that CLEC14A may have more predominant roles in pericyte attachment, or there could be differences in the requirement of CLEC14A or CD93 in pericyte attachment in different tissues.

MMRN2 has been shown to be a substrate for MMP9, although it is unclear whether the subsequent cleaved fragments can still bind to members of the CTLD group 14 family and clearly warrants further investigation 216. Intriguingly, as mentioned previously, CD248 overexpression results in upregulation of MMP9 posing a scenario in which MMRN2 could be processed by MMP9 potentially regulating CD248–MMRN2 binding. Alternatively, CD248‐mediated upregulation of MMP9 may allow cleavage of MMRN2 and detachment of the endothelial–pericyte interaction.

The EGF repeats and mucin‐like regions of both CD93 and thrombomodulin have been reported to have proangiogenic effects. In the case of thrombomodulin, this mitogenic ability was abolished if the CTLD was present on the soluble protein (i.e. including the CTLD, sushi and EGF repeats), although it is unclear whether this also applies for CD93. Nevertheless, one could speculate a scenario where differential proteolytic cleavage of such proteins results in diverse outcomes upon the endothelium and other cell types, allowing fine tuning of cellular events. As discussed previously, there is evidence that the CTLD of thrombomodulin can be shed from the full‐length molecule or from the cleaved ECD. Additionally, there is likely a second cleavage event in the CLEC14A ECD generating a fragment smaller than the full‐length ECD which encompasses the CTLD 211. Multiple proteolytic cleavage events may be true for other CTLD group 14 family members.

### Potential roles in immunosuppression

Angiogenesis and immunosuppression are two tightly regulated processes that often occur in unison. They have been described as parallel processes especially in the context of tumour angiogenesis and tumour immunosuppression 217. Many proangiogenic proteins also mediate immunosuppressive effects upon the vasculature as well as immune cells directly 218. Here we describe some examples of CTLD group 14 family members that elicit immunosuppressive roles. For example, expression of CD248 on naïve T‐cells correlated with decreased cell proliferation. In this setting, CD248 binding to its ligands that are upregulated in tumour angiogenesis (i.e. MMRN2 or fibronectin etc.) may inhibit T‐cell proliferation. Similarly, thrombomodulin expression on the vasculature or perhaps in soluble form can mediate immunosuppressive functions upon binding GPR15 on T‐cells as well as a whole host of other anti‐inflammatory roles as described above. Although high expression of thrombomodulin has been reported by multiple groups in diverse cancer indications, whether thrombomodulin can actually elicit an immunosuppressive function in the context of cancer remains to be elucidated and the finding that low thrombomodulin leads to improved prognosis seems to contradict this theory. CD93 has also been described to trigger anti‐inflammatory events, such as limiting leucocyte migration in peritonitis 170. Other members of the CTLD group 14 family may invoke broader effects upon distinct components of the immune system, and potentially contribute to immunosuppression especially in the context of tumours evading the immune system.

### Potential use as therapeutic targets

So far clinical trials targeting CD248 have proved to be very disappointing, and such agents may only be effective in certain tumour types or may need to be combined with other therapeutics for optimal clinical benefit. However, CD248 targeting antibodies still have promise as therapies for inflammatory or fibrotic diseases where CD248 has a pathological role.

CLEC14A as a therapeutic target of the tumour vasculature has been investigated by many different preclinical strategies using antibodies as well as fragments of its known ligands and even chimeric antigen receptor T‐cells 219, 220. Since it is well established that CLEC14A is expressed on vessels that experience low shear stress and aberrant blood flow, it is conceivable that only nonfunctional tumour vessels will be targeted by such agents. This could prove beneficial as vascular normalisation effects would likely take place, lowering hypoxia, which could lead to better accumulation and delivery of other drugs used in combination such as chemotherapy. Additionally, such CLEC14A targeting could be combined with immunotherapies which rely on infiltration of effector immune cells into the tumour mass, where functional and more ‘normal’ vasculature would likely be advantageous 109.

In studies investigating the use of CLEC14A CTLD specific antibodies, Kim *et al*. tested a human colorectal cancer cell line HCT116 as well as a bevacizumab‐resistant version of this line. Both cell lines showed significant reductions in *in vivo* angiogenesis following treatment with CLEC14A antibodies when these cells were embedded in Matrigel and injected subcutaneously 206. This suggests a possible use for targeting of CLEC14A in patients that have acquired resistance to VEGF blockade. More importantly these findings suggest that although targeting of CLEC14A can reduce VEGF‐dependent angiogenesis in various models, it may also ablate angiogenesis induced by VEGF‐independent pathways. However, the authors did not assess the efficacy of these antibodies in targeting this resistant cell line in tumour xenograft studies; therefore, the tangible benefit of CLEC14A targeting in tumour types resistant to VEGF blockade is yet to be fully established.

Dual targeting of CLEC14A and CD93 was achieved by use of a MMRN2 fragment that contained the CTLD‐binding region (amino acid residues 495–674 in human and 495–678 in mouse) fused to an Fc tag 101. This resulted in a decrease in syngeneic tumour growth *in vivo* and disruptions in angiogenesis *in vitro*. Blocking CLEC14A and CD93 in this manner will likely inhibit endothelial cells binding to endogenous MMRN2 and may even interrupt the fibrogenesis of fibronectin, as has been described with genetic ablation of CD93 167. Furthermore, such targeting strategies may destabilise the binding of CD248 expressing pericytes to the tumour vasculature, although whether this affects pericyte coverage remains to be investigated. There is scepticism in the field in terms of whether such pericyte targeting approaches provide meaningful clinical benefit 221. The use of such dual‐targeting approaches negates the ability of one protein compensating the loss of the other. However, it is important to note that this dual‐targeting MMRN2 fragment Fc fusion protein was expressed directly in the tumour microenvironment by genetically engineered tumour cells. With less restricted expression of the targeting fragment, we cannot rule out the possibility that this agent could display off target effects by binding to other CD93 expressing cell types such as monocytes or B‐cells.

The likelihood of CTLD group 14 family members serving as viable targets in cancer therapy will ultimately depend on the expression profile of these proteins, which if not tumour or tumour vasculature specific could result in toxicity‐related issues in patients. In this regard, a seminal paper investigating targeting the tumour endothelial marker and immunomodulatory molecule CD276 (also referred to as B7‐H3) described that the most important determining factor for avoiding toxicity is in fact level of expression 222, 223 **.** Indeed, even though CD276 displays a widespread expression pattern in mouse and human tissues, the fact that it is so highly expressed by tumour cells and the associated tumour vasculature, resulted in ADCs against CD276 only having substantial effects upon the tumour microenvironment. In light of this data, experiments that determine levels of target protein expression will likely become paramount. Relatedly, low‐affinity, high avidity therapeutic agents could be used against targets that are highly expressed on tumour‐associated tissue but still expressed lowly on normal tissue; in this way, agents would preferentially bind to highly expressing cell types. This approach was demonstrated with low‐affinity, high avidity HER‐2/CD3‐binding bispecific agents that redirect T‐cells towards breast cancer cells 224. These high‐avidity bispecific antibodies induced negligible effects on *in vivo* tumour models expressing low levels of HER‐2 but successfully eradicated high‐expressing tumour lines, suggesting that normal tissues expressing HER‐2 at low levels may be avoided and toxicity minimised.

As mentioned previously, the cleavage of the CTLD group 14 family members may negatively impact antibody targeting therapies, as the soluble forms may sequester the antibodies in the blood rendering them incapable of binding to the cell surface receptors. However, this issue will likely be addressed in preclinical models and presumably be overcome in phase I dose escalation studies of CTLD group 14 family targeted agents.

### High priority areas of future research

There are many unanswered questions relating to the physiological and pathological functions of this family of molecules, especially for CD248, CD93 and CLEC14A. In particular, information on intracellular signalling events is currently lacking.

It is currently unknown whether CLEC14A and CD93 share other extracellular binding partners as is the case for MMRN2. Likewise, other CTLD group 14 members may share additional binding proteins as has been shown for HSP70‐1 binding both CLEC14A and thrombomodulin 25, 207. The region of human CLEC14A CTLD that engages HSP70‐1A exhibits 25.9% sequence identity to thrombomodulin, 29.6% identity to CD93 and 33.3% to CD248; however, the domain important for the thrombomodulin‐HSP70‐1 interaction has not yet been defined. Clearly further binding experiments to extensively characterise interactions between other CTLD group 14 family members and newly described ligands will need to be conducted.

It is uncertain whether CLEC14A and CD93 compete for binding with MMRN2 or whether they have independent or similar roles. Also, the signalling outcomes following MMRN2 binding to CLEC14A, CD93 or CD248 are not fully established. CLEC14A and CD93 have been postulated to have redundant roles in zebrafish angiogenesis but not vasculogenesis 225. Simultaneous knockout of both CLEC14A and CD93 led to more severe defects in intersegmental vessel formation compared with single‐gene knockouts, now reported by two different groups 195. VE‐cadherin expression was absent in vessels that lacked CLEC14A and CD93, suggesting abnormalities in endothelial cell–cell adhesion, when VE‐cadherin was replaced this rescued the detrimental phenotype. Knockdown of CD93 has also been shown to reduce VE‐cadherin levels in HUVEC 156, although phenotypic outcomes following double knockdown of both CLEC14A and CD93 have not yet been reported in mammalian cell types. Currently there are no data on whether CLEC14A and CD93 display redundancy in mammals, and a double KO mouse would begin to address this important question.

Finally, as CLEC14A and CD93 have been described as proteins highly expressed in the tumour vasculature, with roles in angiogenesis, it remains to be investigated whether these molecules are also expressed and have roles in other routes in which a tumour can acquire a blood supply. Two such mechanisms involve (a) vessel co‐option, whereby tumour cells hijack the existing vasculature in highly vascularised organs such as the lung, liver and brain 226, or (b) vascular mimicry, where tumour cells can exhibit endothelial‐like properties and form functional vessel like structures 227.

## Concluding remarks

The CTLD group 14 is a family of molecules with diverse roles in the vasculature, inflammation as well as tumour progression. The increasing interest in these molecules including elucidation of their normal biology as well as their potential as therapeutic targets in cancer will likely continue to be explored in the future.

## Conflict of interest

KAK and RB are inventors on patents WO/2016/116760 entitled ‘Inhibitors of the interaction between CLEC14A and Multimerin‐2 for inhibition of angiogenesis’ and the related filed patents GB1612860.5, GB1612534.6 and GB1702926.5.

## Author contributions

KAK designed and wrote the manuscript as well as directed analysis of the Tabula Muris data. JLM analysed the Tabula Muris data and edited and reviewed the manuscript. FM and RB aided in writing, editing and review of the manuscript.
